# Mortality and Major Morbidity of Very-Low-Birth-Weight Infants in Germany 2008–2012: A Report Based on Administrative Data

**DOI:** 10.3389/fped.2016.00023

**Published:** 2016-03-22

**Authors:** Elke Jeschke, Alexandra Biermann, Christian Günster, Thomas Böhler, Günther Heller, Helmut D. Hummler, Christoph Bührer, for the Routine Data-Based Quality Improvement Panel

**Affiliations:** ^1^Wissenschaftliches Institut der Ortskrankenkassen, Berlin, Germany; ^2^Medizinischer Dienst der Krankenkassen Baden-Württemberg, Karlsruhe, Germany; ^3^Institut für Qualität und Transparenz im Gesundheitswesen, Berlin, Germany; ^4^Section Neonatology/Pediatric Intensive Care, Ulm University Children’s Hospital, Ulm, Germany; ^5^Department of Neonatology, Charité University Medical Center, Berlin, Germany

**Keywords:** preterm infant, mortality, morbidity, risk factor, predictive power

## Abstract

**Background:**

Expectant parents of very preterm infants, physicians, and policy makers require estimates for chances of survival and survival without morbidity. Such estimates should derive from a large, reliable, and contemporary data base of easily available items known at birth.

**Objective:**

To determine short-term outcome and risk factors in very-low-birth-weight preterm infants based on administrative data.

**Methods:**

Anonymized routine data sets transmitted from hospital administrations to statutory health insurance companies were used to assess survival and survival free of major morbidities in a large cohort of preterm infants in Germany.

**Results:**

After exclusion of infants with lethal malformations, there were 13,147 infants with a birth weight below 1,500 g admitted to neonatal care 2008–2012, of whom 1,432 infants (10.9%) died within 180 days. Estimated 180 days survival probabilities were 0.632 (95% confidence interval 0.583–0.677) for infants with 250–499 g birth weight, 0.817 (0.799–0.834) for 500–749 g, 0.931 (0.920–0.940) for 750–999 g, 0.973 (0.967–0.979) for 1,000–1,249 g, and 0.985 (0.981–0.988) for 1,250–1,499 g. Estimated probabilities for survival without major morbidity (surgically treated intraventricular hemorrhage, necrotizing enterocolitis, intestinal perforation, or retinopathy) were 0.433 (0.384–0.481) for 250–499 g, 0.622 (0.600–0.643) for 500–749 g, 0.836 (0.821–0.849) for 750–999 g, 0.938 (0.928–0.946) for 1,000–1,249 g, and 0.969 (0.964–0.974) for 1,250–1,499 g, respectively. Prediction of survival and survival without major morbidities was moderately improved by adding sex, small for gestational age, and severe or moderate congenital malformation, increasing receiver operating characteristic areas under the curve from 0.839 (0.827–0.850) to 0.862 (0.852–0.874) (survival) and from 0.827 (0.822–0.842) to 0.852 (0.846–0.863) (survival without major morbidities), respectively.

**Conclusion:**

The present analysis encourages attempts to use administrative data to investigate the association between risk factors and outcome in preterm infants.

## Introduction

In threatened preterm delivery at the threshold of viability, estimating the chances for survival and survival without major morbidities of very preterm infants becomes pivotal when parents and physicians are faced with decisions to begin or withhold treatment. Furthermore, it is also important when designing interventional trials or for making adjustments in quality improvement efforts that compare hospital performance. Outcome of preterm infants largely depends on birth weight and gestational age, and these variables remain the most commonly used denominators in population-based reports (1–9). National recommendations to initiate or withhold treatment at the threshold of viability are mostly based on gestational age (10), while the European Resuscitation Council discourages resuscitative efforts in infants below 23 weeks gestational age or those with a birth weight below 400 g (11). The likelihood of a favorable outcome may be predicted more accurately when further factors present at birth are taken into account (12). The U.S. National Institute of Child Health and Human Development (NICHD) Neonatal Research Network, analyzing data of 4,446 preterm infant with a birth weight of 401–1,000 g admitted 1998–2003 to 19 U.S. hospitals, has demonstrated that prediction of survival and survival without major neurodevelopmental impairment can be better estimated by consideration of five *a priori* selected factors (birth weight, gestational age, sex, exposure to antenatal corticosteroids, multiple gestation) than with use of gestational age alone (13). Two subsequent population-based studies in Victoria, Australia (2005, 114 infants) (14) and California, USA (2005–2008, 4,527 infants) (15) confirmed that using the five variables gave superior power to predict mortality than using gestational age only, although prediction of mortality for outborns was poor. The Canadian Neonatal Network has provided graphical representations of the probabilities of survival and survival without major morbidities calculated from gestational age, birth weight, and sex of 17,148 preterm infants admitted 2003–2008 to all major level III neonatal intensive care units in Canada, excluding only those with lethal congenital anomalies, primary palliation, missing values, or extreme outliers (16). The predictive power of the model was only marginally improved upon addition of the further variables, such as antenatal steroids, multiple gestation, mode of delivery, and maternal smoking, while it increased with inclusion of variables reflecting the clinical course during the first 24 h of life (17).

Rates of survival and survival without major morbidities vary considerably between countries, and they tend to improve over time (4, 7, 8, 18–20). Thus, there is a need to analyze actual regional or national data to meet the demand for contemporaneous outcome estimates. Here, we report rates of 180 days survival and survival free of major morbidities in preterm infants below 1,500 g birth weight in Germany 2008–2012 based on birth weight and assessed the impact of risk factors on outcome of these infants.

## Materials and Methods

In Germany, family health insurance is mandatory for all employees with an annual income below 53,550 €, including recipients of unemployment benefits or welfare. In total, more than 90% of the population are covered by this statutory health insurance. While individuals can choose freely among 132 statutory health insurance providers, regardless of age, morbidities, income, or type of employment, there are 11 large regional health care funds covering about 33% of the persons insured. These large regional health care funds jointly run a scientific institute entrusted with collecting routine patient data sets sent from the hospital administrations to these 11 statutory health insurance companies to obtain reimbursement. The data sets that also contain the date the health insurance ends because the patient has died or changed the company are analyzed in an anonymized fashion to support quality improvement initiatives and provide health policy makers with decision aids.

We present an analysis of the data sets of live-born preterm infants born between January 1, 2008 and December 31, 2012 with a birth weight of 250–1,499 g who were admitted to hospital care within the first 24 h life and covered by one of the 11 regional health care funds. The routine data sets transmitted from the hospital administrations to the insurance companies to receive reimbursements include diagnoses (ICD-10), major procedures (German version of ICPM), sex, and weight on admission (for infants below 1 year of age).

We analyzed only data of infants admitted to neonatal care, any stillborn infants and intrapartum deaths were excluded, as were infants with lethal malformations. Malformations and congenital diseases were considered lethal, severe, or moderate according to their 180 days mortality rate in very-low-birth-weight infants calculated from the regional health care fund data base (>66%, lethal: thanatophoric dysplasia, anencephaly, bilateral renal agenesis, Potter sequence, autosomal-dominant polycystic kidney disease, bladder exstrophy, trisomy 13 or 18, tri- or polyploidy, hypoplastic left heart syndrome, double outlet right ventricle, aortic atresia, non-immune fetal hydrops; 33–66%, severe: spina bifida, urea cycle defects, congenital diaphragmatic hernia, common arterial trunk, discordant ventriculoarterial connection, double inlet ventricle, total anomalous pulmonary venous connection; 16.5–32.9%, moderate: esophageal atresia, omphalocele, non-autosomal-dominant polycystic kidney disease, tetralogy of Fallot, aortic coarctation, other congenital heart diseases). *A priori* selected predictor variables were birth weight (entered as categorical variable per 100 g increments), severe and moderate malformations, small for gestational age, sex, and multiple gestation (8, 12, 13, 21). Endpoints were mortality up to 180 days of life (during the first hospitalization, any subsequent hospitalizations, or at home) and any surgical interventions performed during the first 180 days of life for high-grade intraventricular hemorrhage (IVH), necrotizing enterocolitis (NEC)/spontaneous intestinal perforation, or retinopathy of prematurity (ROP). Any brain surgery in infants with IVH, such as shunt placement or endoscopic drainage, was considered surgical intervention, as was drainage or laparotomy in infants with NEC or spontaneous intestinal perforation. For ROP, cryotherapy, laser treatment, and intravitreal injections of any of the anti-VEGF drugs bevacizumab, ranibizumab, aflibercept, or pegaptanib were considered surgical interventions.

Statistical analyses were carried out using STATA (Version 11.2, Stata Corp, College Station, TX, USA). Kaplan–Meier analysis was used to describe survival up to 180 days and survival without major neonatal morbidity, with strata being compared by the log-rank test. The impact of predictor variables was assessed with adjusted odds ratios (OR) in multiple logistic regression analyses. To describe the power to predict survival and survival without major neonatal morbidity, receiver operating characteristics (ROC) curves were generated, and the areas under the curve with 95% confidence intervals were calculated.

The study received institutional review board approval (Ethikkommission der Charité Universitätsmedizin Berlin, # EA2/136/14). No consent was required as this is a retrospective analysis of anonymized data collected according to federal law.

## Results

A total of 13,303 infants covered by the regional statutory health insurance companies were born between January 1, 2008 and December 31, 2012 with a birth weight between 250 and 1,499 g. Of these, 156 were excluded (stillborn, *n* = 17; lethal malformations: *n* = 128, twins with identical numbers: *n* = 17). The characteristics of the 13,147 patients included are given in Table 1. There were 143 cases (1.09%) with incomplete data sets due to a change of the insurance company within 180 days after birth. There were a higher percentage of girls (51.2 vs. 48.7%, *p* = 0.0216) and small-for-gestational-age babies (21.4 vs. 18.4%, *p* = 0.0004) in infants with a birth weight of 250–749 g, as compared to infants of 750–1,499 g, while the opposite was observed for multiples (11.4 vs. 20.7%, *p* = 0.0001).

**Table 1:** **Patients’ characteristics stratified by birth weight**.

	Total *N* (%)	250–499 g *N* (%)	500–749 g *N* (%)	750–999 g *N* (%)	1000–1249 g *N* (%)	1,250–1,499 g *N* (%)
Number	13,147 (100)	673 (100)	2,147 (100)	2,764 (100)	3,061 (100)	4,502 (100)
Girls	6,473 (49.2)	353 (52.5)	1,090 (50.8)	1,351 (48.9)	1,478 (48.3)	2,201 (48.9)
Small for gestational age	2,502 (19.0)	216 (32.1)	387 (18.0)	434 (15.7)	566 (18.5)	899 (20.0)
Multiple gestation	2,456 (18.7)	94 (14.0)	328 (15.8)	498 (18.0)	556 (18.2)	980 (21.8)
Severe malformations	53 (0.4)	3 (0.5)	7 (0.3)	6 (0.2)	14 (0.5)	23 (0.5)
Moderate malformations	128 (1.0)	13 (1.9)	20 (0.9)	26 (0.9)	23 (0.8)	46 (1.0)

**Figure 1:**
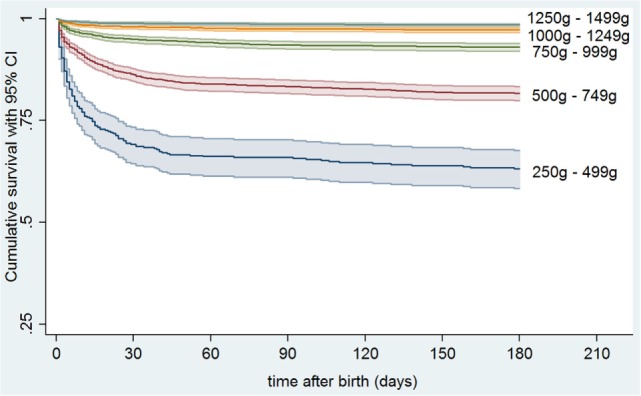
**Kaplan–Meier estimates for survival up to 180 days of life in preterm infants stratified by birth weight**.

Mortality within 180 days of life was 10.9% (1,432/13,147) in the total group, and 22% (1,229/5,584) in infants below 1,000 g birth weight (Table 2). Estimated probabilities (95% confidence intervals) of 180 days survival were 0.632 (0.5831–0.677) for infants with a birth weight of 250–499 g, 0.817 (0.799-0.834) for 500–749 g, 0.931 (0.920–0.940) for 750–999 g, 0.973 (0.967–0.979) for 1,000–1,249 g, and 0.985 (0.981–0.988) for 1,250–1,499 g, respectively (Figure 1). On average, 202 (14.1%) of deaths occurred between 30 and 180 days of life (Table 2), of which 35 (2.4%) were observed after discharge.

**Table 2:** **Mortality and major morbidity stratified by birth weight**.

	Total *N* (%)	250–499 g *N* (%)	500–749 g *N* (%)	750–999 g *N* (%)	1,000–1,249 g *N* (%)	1,250–1,499 g *N* (%)
Numbers (percentage)	13,147 (100)	673 (100)	2,147 (100)	2,764 (100)	3,061 (100)	4,502 (100)
**Mortality**						
• Within 30 days of life	1,230 (9.4)	394 (58.4)	477 (22.2)	195 (7.1)	88 (2.9)	76 (1.7)
• Within 180 days of life	1,432 (10.9)	417 (62.0)	565 (26.3)	247 (8.9)	110 (3.6)	93 (2.1)
**Major neonatal morbidity**						
• Intraventricular hemorrhage, surgical treatment within first 180 days of life	296 (2.3)	13 (1.9)	78 (3.6)	106 (3.8)	59 (1.9)	40 (0.9)
• Necrotizing enterocolitis or intestinal perforation, surgical treatment within first 180 days of life	474 (3.6)	43 (6.4)	199 (9.3)	142 (5.1)	57 (1.9)	33 (0.7)
• Retinopathy of prematurity, local treatment (cryo, laser, anti-VEGF injection) within first 180 days of life	397 (3.0)	60 (8.9)	227 (10.6)	88 (3.2)	18 (0.6)	4 (0.1)
**Alive at 180 days**						
• Without major morbidity	10,807 (82.2)	174 (25.9)	1,200 (55.9)	2,257 (81.7)	2,841 (92.8)	4,335 (96.3)
• With one major morbidity	782 (5.9)	64 (9.5)	322 (15.0)	222 (8.0)	100 (3.3)	74 (1.6)
• With more than one major morbidity	126 (1.0)	18 (2.7)	60 (2.8)	38 (1.4)	10 (0.3)	0 (0.0)
• Total	11715 (89.1)	256 (38.0)	1582 (73.7)	2517 (91.1)	2957 (96.6)	4409 (97.9)

**Figure 2:**
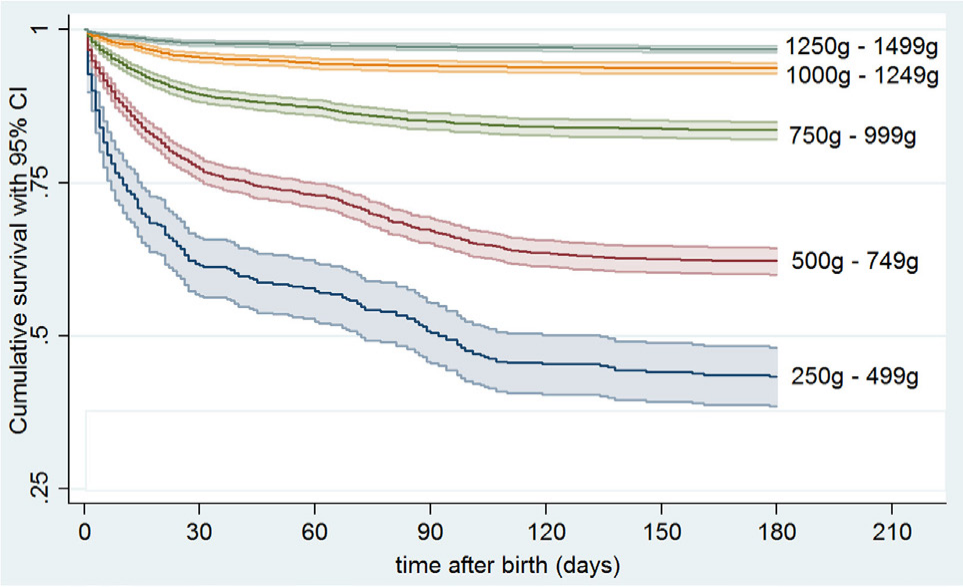
**Kaplan–Meier estimates for survival up to 180 days of life without major morbidity (surgically treated intraventricular hemorrhage, necrotizing enterocolitis/intestinal perforation, or retinopathy of prematurity) in preterm infants stratified by birth weight**.

**Figure 3:**
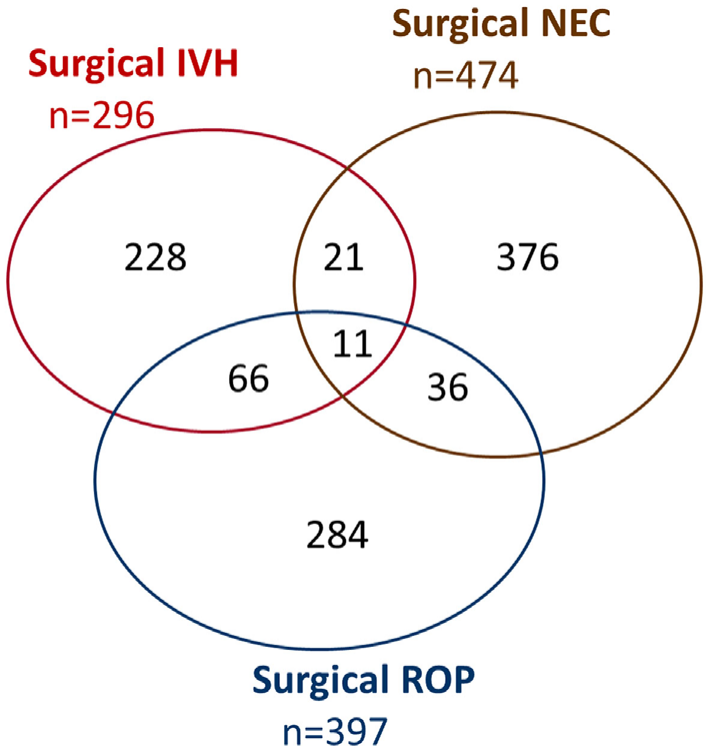
**Venn diagram of major neonatal morbidities with surgical intervention (all infants)**. IVH, intraventricular hemorrhage; NEC, necrotizing enterocolitis (including any intestinal perforation); ROP, retinopathy of prematurity.

**Figure 4:**
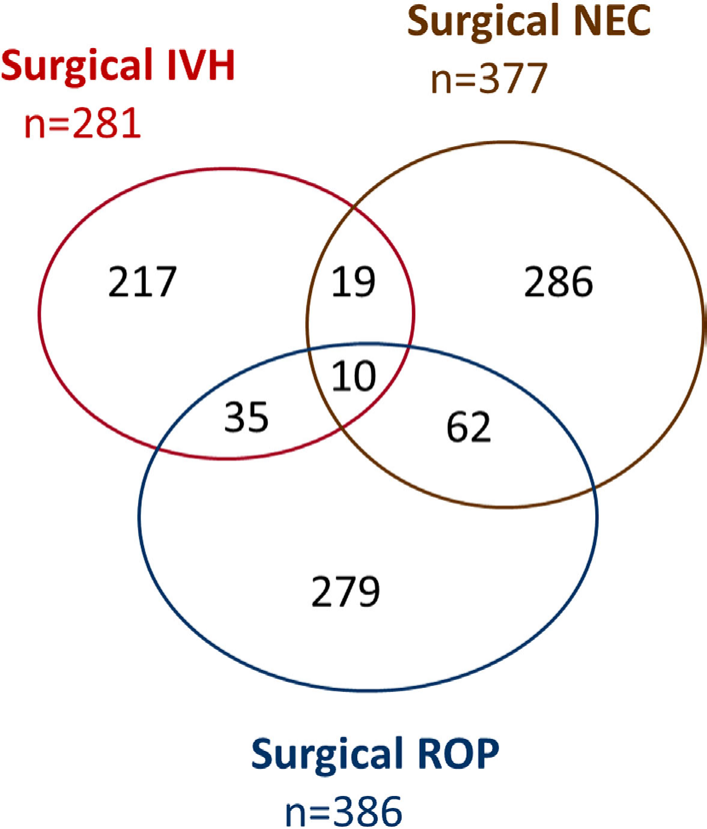
**Venn diagram of major neonatal morbidities with surgical intervention (survivors only)**. IVH, intraventricular hemorrhage; NEC, necrotizing enterocolitis (including any intestinal perforation); ROP, retinopathy of prematurity.

Major neonatal morbidities with surgical interventions occurred in 1022/13,147 (7.8%) infants. Estimated probabilities of 180 days survival without major morbidity were 0.433 (0.384–0.481) for 250–499 g, 0.622 (0.600–0.643) for 500–749 g, 0.836 (0.821–0.849) for 750–999 g, 0.938 (0.928–0.946) for 1,000–1,249 g, and 0.969 (0.964–0.974) for 1,250–1,499 g, respectively (Figure 2). Overall, there were 888 (6.8%), 123 (0.9%), and 11 (0.08%) infants suffering from one, two, or three morbidities, respectively (Figure 3), and 782 (6.7%), 116 (1.0%), and 10 (0.09%) in survivors (Figure 4). Among the 474 infants who had undergone surgery for NEC/intestinal perforation, there were 97 deaths (20.5%), as compared to 15/296 (5.1%) after surgery for IVH, and 11/397 (2.7%) after intervention for ROP.

Multiple logistic regression analysis was used to estimate the impact of the *a priori* selected predictor variables birth weight (per each 100-g increment, with the group of infants with a birth weight of 1,400–1,499 g serving as reference), sex, small for gestational age, multiple gestation, moderate, and severe congenital malformations, on survival and survival without major morbidity (Table 3). Except for multiple gestation, all of the *a priori* selected variables retained a statistically significant association with both death and death or survival with major morbidity upon multiple regression analysis.

**Table 3:** **Multivariate predictor variables for death or death/survival with major morbidity**.

Variable	OR death	5–95% CI	OR death or survival with major morbidity	5–95% CI
**Birth weight (g)**				
<300	2026.27	265.06–15,489.06	578.61	131.77–2540.60
300––399	269.96	166.15–438.61	288.90	173.14–482.06
400–499	104.05	73.11–148.09	105.93	77.58–144.65
500–599	42.03	30.11–58.66	47.25	35.85–62.28
600–699	20.15	14.51–27.97	26.63	20.53–34.54
700–799	10.76	7.68–15.08	12.15	9.34–15.79
800–899	5.62	3.93–8.06	6.95	5.30–9.11
900–999	4.02	2.82–5.73	4.99	3.83–6.50
1,000–1,099	3.37	2.20–5.17	3.19	2.29–4.46
1,100–1,199	1.92	1.26–2.93	2.44	1.80–3.31
1,200–1,299	1.39	0.90–2.15	1.24	0.88–1.74
1,300–1,399	1.35	0.86–2.11	1.08	0.75–1.56
1,400–1,499	1	(Reference)	1	(Reference)
Male	1.59	1.40–1.82	1.65	1.48–1.85
Small for gestational age	0.24	0.19–0.29	0.23	0.19–0.28
Multiple	1.07	0.90–1.27	1.08	0.94–1.25
Moderate malformation	1.68	1.11–2.55	1.55	1.05–2.29
Severe malformation	17.34	9.52–31.58	11.59	6.29–21.37

**Figure 5:**
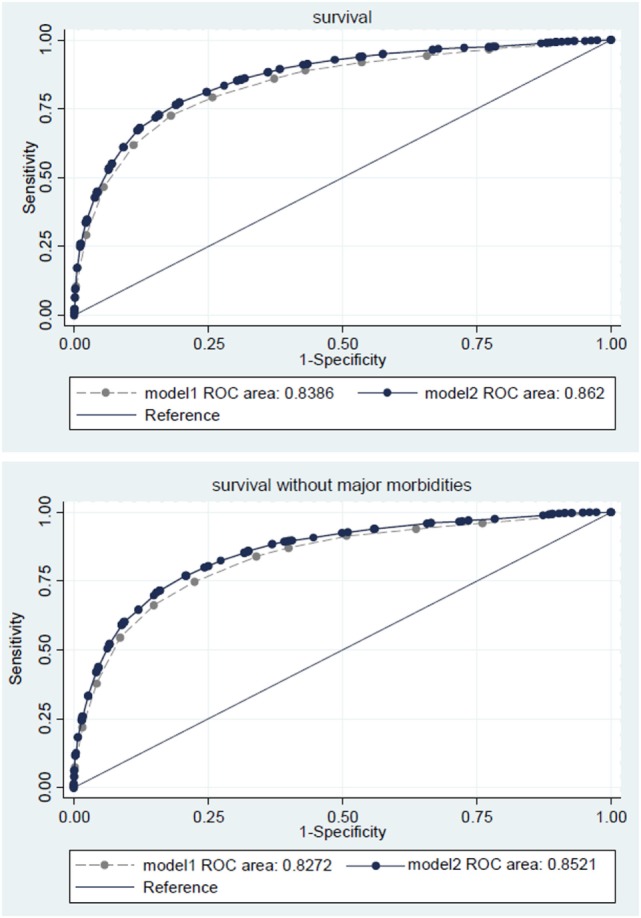
**Receiver operating characteristic (ROC) curve for survival (upper panel) or survival without major morbidities (lower panel) based on birth weight (model 1, dashed lines) or birth weight, small for gestational age, sex, multiple gestation, severe or moderate malformations (model 2, solid lines)**.

The areas under the ROC to predict survival and survival without major morbidity were 0.862 (0.852–0.874) and 0.852 (0.846-0.863) taking the *a priori* selected variables into account, as compared to 0.839 (0.827–0.850), and 0.827 (0.822–0.842) when only birth weight was used (Figure 5). ROC areas were smaller when looking only at infants below 1,000 g birth weight [full model: 0.800 (0.785–0.815) for survival, 0.785 (0.772–0.797) for survival without major morbidity; birth weight only: 0.771 (0.755–0.786) for survival, 0.746 (0.732–0.760) for survival without major morbidity].

## Discussion

This analysis reports an overall 180 days survival rate of preterm infants with a birth weight <1,500 g admitted to neonatal intensive care units in Germany 2008–2012 of 89.1%, with 14.1% of deaths occurring between 30 and 180 days of life. Surgically treated major morbidities were observed in 7.7% of all very-low-birth-weight infants, and 7.8% of survivors, respectively. Rates of survival strongly declined alongside decreasing birth weight, from 97.9% in 1,250–1,499 g birth weight infants to 38% in infants below 500 g birth weight, while the rates of surgically treated major morbidities in survivors increased from 1.7 to 32%.

We compared our data with published data of a cohort of 2,207 infants below 1,500 g birth weight without lethal malformations treated in 2010 in a network of 46 tertiary care neonatal units in Germany (22). The published survival rate prior to discharge in this network-based cohort (90.6%) was virtually identical to the 30 days survival rate observed in the 2008–2012 data set. We also compared our data with those of cohorts from other countries. For infants below 1,000 g birth weight admitted to neonatal care, reported survival at 2 years was 70.8% (613/866) in a Swiss population-based cohort 2000–2008 (7), as compared with 78% (4,355/5,584) 180 days survival in the German 2008–2012 cohort. Unfortunately, most published reports do not provide survival rates by birth weight, and survival figures are given at differing time points – prior to discharge (4, 19), at 1 year (18), or at 2 years of life (9). Some crude comparisons, however, are made possible by the similarity of average birth weights (730–750 g) in cohorts of preterm infants <27 weeks gestation (730–750 g) in Sweden (18), England (4), France (19), and the United States (9), and infants <1,000 g birth weight who are subject of this study. The published 1-year or 2-year survival rates of the infants admitted to neonatal intensive care were 78.1% (497/636) in the Swedish EXPRESS cohort (2004–2007) and 65.0% (2,630/4,046) in the American NICHD network (2006–2011), respectively, while survival to discharge was 61.7% (1,041/1,686) in the English EPICure2 (2006) cohort and 69.0% (552/800) in the French EPIPAGE2 (2011) cohort. Of note, these outcome data are influenced by physicians’ and parents’ choices on which infants to admit to neonatal care. We assume that the lower percentages of boys and multiples we observed in preterm infants with 250–749 g birth weight admitted to neonatal care, as compared to infants with 750–1,499 g birth weight, are most likely a reflection of such choices.

Outcome was strongly related to birth weight, with risks for mortality or major morbidity on average doubling for each 20–25% decrease in birth weight. The impact of birth weight and the other *a priori* selected variables on death and death and major morbidity combined was remarkably similar. Multiple gestation was not linked to poor outcome, an observation made for recent cohorts also in Australia and New Zealand (23). Small for gestational age was associated with reduced ORs for poor outcome as the investigation used birth weight as primary variable, the opposite would have been observed with gestational age as primary variable. While sex, small for gestational age, and malformations were independently associated with outcome, estimating chances for survival and survival without surgical morbidities based on birth weight alone was only slightly improved by the addition of these further variables. This is in line with previous observations, showing that birth weight alone was only slightly inferior than the clinical risk index for babies calculated from birth weight, gestational age, congenital malformations, base excess, and oxygen requirements during the first 12 h of life in predicting death or neurodevelopmental impairment at 1 year of age (24). The predictive power, as measured by the area under the ROC curve, was similar to that of similar published algorithms taking only variables into account that are known at birth (13, 16). Unfortunately, the lack of certain items in the data set available prevented any direct head-to-head comparison.

This study has several strengths and weaknesses. It is based on a large, contemporary cohort, with limited selection bias or loss to follow-up. Well-to-do individuals, however, are underrepresented in the cohort of the study, as self-employed persons and employees with an annual income above 53,550 € may choose private health insurance instead of statutory health insurance (about 10% of the population). This drawback is to be balanced against the risk of recruitment bias in prospective clinical trials, especially those that require antenatal parental consent (25), the risk of referral bias for data from networks of highly specialized groups of neonatal intensive care units, and bias introduced into observational studies by uneven loss to follow-up, with an excess of children from disadvantaged families who are not being evaluated face to face (26).

Our investigation was based on data from patients rather than cases, whereas events occurring after discharge or transfer to another hospital are being missed in unit-based network and quality improvement data sets. In contrast to other studies, the 180 days window allowed to take events after discharge or transfer to another hospital into account, unless there was a change in the company providing health care insurance. Late deaths are an important issue, especially in very small infants, with 13.3% of deaths occurring after 30 days of life in infants below 1,000 g birth weight. For comparison, 17.3 and 19.8% of all deaths occurred beyond 28 days of life in the American NICHD (8) and English EPICure2 cohorts (4), respectively.

As a major weakness, some variables of great interest to clinicians are missing, particularly gestational age at birth, antenatal steroids, mode of delivery, and inborn/outborn status. A merger of the anonymized infant administrative data sets, as used in this study, with maternal records is being hampered by current rules governing privacy protection. The variable “small for gestational age” partly made up for the missing variable “gestational age at birth,” also avoiding the multicollinearity problem when birth weight and gestational age are entered simultaneously as continuous measures. The variable “antenatal steroids” has lost statistical impact as a result of the high rates of antenatal steroids and exogenous surfactant replacement therapy (27). As data verification or central review is not possible with anonymized data sets, we chose simple endpoints each representing an irreversible event marked with a calendar date (mortality, surgery for neonatal diseases) to reduce the risk of reporting bias. This also allowed for Kaplan–Meier analyses not possible with time varying variables that wax and wane during the clinical course, such as ROP or bronchopulmonary dysplasia.

High-grade IVH, high-grade ROP, and NEC requiring surgery have been shown to be strong predictors of long-term neurodevelopmental impairment (7, 28–35). The high inter-rater variability of disease severity for IVH, ROP, or NEC argues against their use as outcome measures, unless the severity is validated by a procedure. IVH, ROP, and NEC are diseases striking preterm infants with various intensities, and the presence of surgical interventions to treat these diseases was used to delineate severe from mild stages. This assumption may not be true in all cases, and counting only morbidity linked with a surgical intervention likely results in overestimation of survival without severe morbidity. However, as surgical procedures increase reimbursements paid to hospitals, the use of interventions as an outcome variable entails little risk of underreporting, in contrast to the mere diagnoses IVH, ROP, and NEC that are liable to down-coding. Abnormal cranial ultrasound findings may give misleading results, considering the high interobserver variability in assessment of cranial ultrasound findings (36, 37) and the poor correlation between high-grade IVH, as depicted by early cranial ultrasound, and neurodevelopmental impairment (37, 38). The association between NEC and poor neurodevelopmental outcome appears to be confined to infants with NEC-related abdominal surgery (29).

Data entered into any type of registry are liable to inadvertent mistakes, biased coding, or even wilful deceit. When estimating the risk of false entries, there are several questions to be addressed: (1) How reliable is the information given to the person who enters the data? (2) How likely are errors based on negligence? (3) Are there incentives for up-coding, down-coding, or omission of data? For these reasons, we chose to not include bronchopulmonary dysplasia as an outcome variable in this analysis, as it lacks an unambiguous marker to disassociate mild from severe forms unless strict definitions are being employed. Some degree of disordered lung development is common in very preterm infants, and it largely depends on the exact criteria used whether or not infants are classified as having bronchopulmonary dysplasia (39). For mortality, which is at center stage of this analysis, administrative data of insurance companies are probably better than data from anywhere else, as the death of a patient also implies the end of the insurance contract. By contrast, an analysis of the mandatory German neonatal registries (used to compare the performance of individual units) showed that more than 30% of extremely preterm infants born alive (by official birth registry) were not included in these registries (40).

Administrative data have been used successfully for as diverse purposes as to estimate live delivery rates after tubal sterilization reversal (41), to compare the risk of preterm birth among women living with and without HIV infection (42), to calculate the risk of hospital readmission among infants with neonatal abstinence syndrome (43), or to examine patterns of pediatric emergency department visits (44, 45). The present analysis encourages attempts to use administrative data also to investigate the association between risk factors and outcome in preterm infants. When it comes to deciding which data to include in such administrative data sets, clinicians should be consulted to facilitate such investigations.

## Author Contributions

EJ, AB, CG, TB, GH, HH, and CB all contributed to the conception and design of this analysis. EJ, AB, and CG provided the statistical analysis, EJ and CB drafted the first manuscript that was revised by AB, CG, TB, GH, and HH and finally approved by all authors.

## Conflict of Interest Statement

The authors declare that this work was done in the absence of any commercial or financial relationships that could be construed as a potential conflict of interest.

## Abbreviations

ICD, international classification of diseases; ICPM, international classification of procedures in medicine; IVH, intraventricular hemorrhage; NEC, necrotizing enterocolitis; NICHD, National Institute of Child Health and Human Development; OR, odds ratio; ROC, receiver operator characteristics; ROP, retinopathy of prematurity; VEGF, vascular endothelial growth factor.
